# Facial nerve paralysis as initial symptom of langerhans cell histiocytosis^☆^^☆☆^

**DOI:** 10.1016/j.bjorl.2016.05.006

**Published:** 2016-06-16

**Authors:** Onur Ismı, Rabia Bozdogan Arpaci, Anıl Ozgur, Elvan Caglar Citak, Neslihan Eti, Tugce Puturgeli, Yusuf Vayisoglu

**Affiliations:** a University of Mersin, Faculty of Medicine, Department of Otorhinolaryngology, Mersin, Turkey; b University of Mersin, Faculty of Medicine, Department of Pathology, Mersin, Turkey; c University of Mersin, Faculty of Medicine, Department of Radiology, Mersin, Turkey; d University of Mersin, Faculty of Medicine, Department of Pediatric Oncology, Mersin, Turkey

## Introduction

Langerhans cells (LCs) are antigen presenting dendritic cells found in epidermis, respiratory and genital epithelia.^1^ In 1953, Lichenstein introduced the term eosinophilic granuloma, Hand–Schuller–Christian disease and Letterer–Siwe disease which are characterized by clonal proliferation of LCs. In 1985 Philadelphia Workshop of the Histiocyte society adapted the term langerhans cell histiocytosis (LCH) to gather these different synonyms under one term.^2^

LCH is a very rare disease seen in approximately one case per 2 million children, males are more frequently affected than females, and presenting age varies between a few months to 15 years with a peak incidence of 1–4 years.^1^ This rare entity can involve anywhere in the body as a solitary lesion or multisystem involvement, temporal bone involvement occurs in 19–25% of cases, with bilateral involvement in 1/3 of these ones.^1^ In the case of ear involvement most common symptoms are chronic ear discharge resistant to antibiotic treatment, postauricular swelling, hearing loss, otalgia and temporo-zygomatic mass. Facial nerve paralysis is a very rare finding in LCH with an average incidence of 2.8%.^3^

In this case presentation, we demonstrated a LCH patient with facial paralysis as initial symptom.

## Case report

Written consent was taken from the patient's parents for this case presentation.

**Figure 1:**
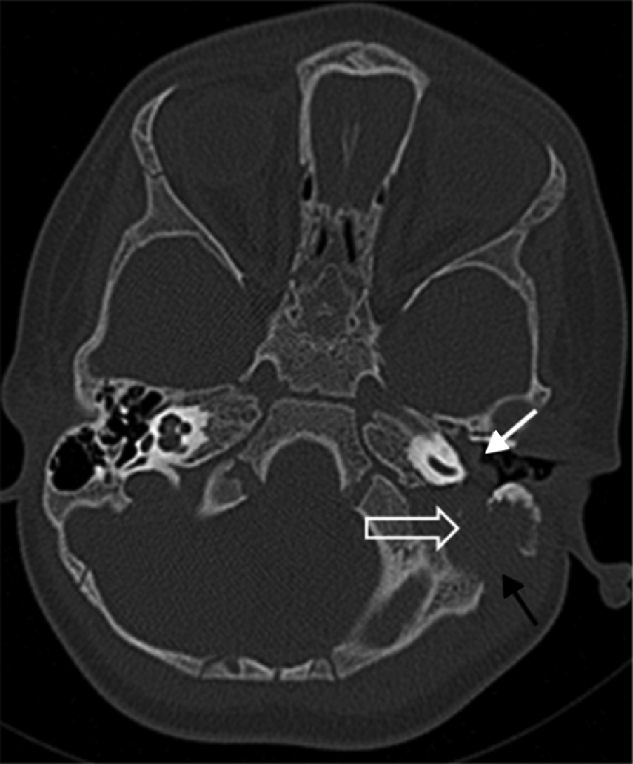
Axial computed tomography image at bone window shows a mass (open arrow) within the left mastoid air cells with an extension into the middle ear cavity (white arrow). Note the associated cortical erosion at the mastoid bone (black arrow).

**Figure 2:**
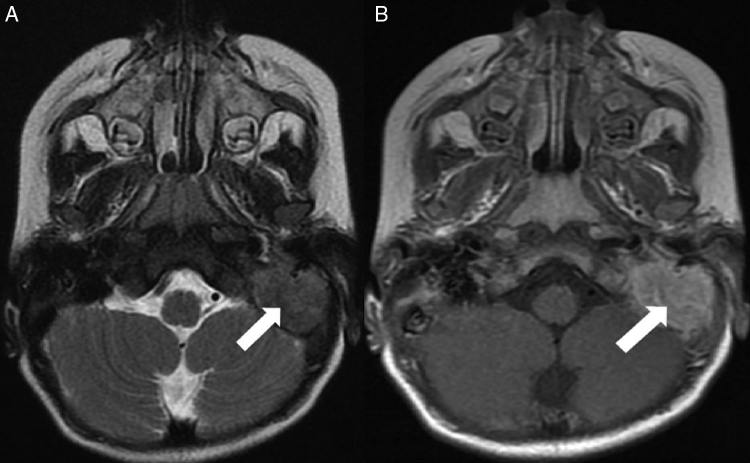
Axial T2-weighted magnetic resonance image (A) demonstrates a hypointense mass (arrow) occupying the left mastoid cavity. Postcontrast T1-weighted image; (B) reveals homogeneous enhancement within the mass (arrow).

**Figure 3:**
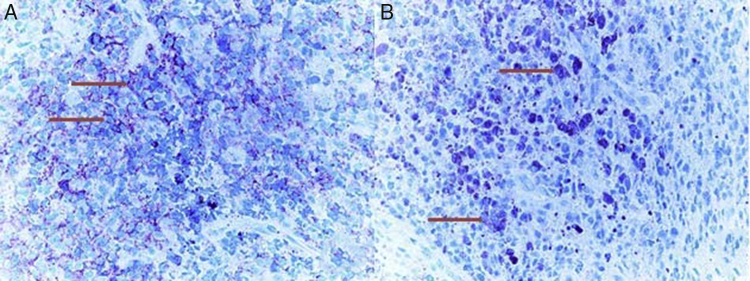
(A) Arrows show tumor cells which were positively stained with CD1a (CD1a, 400×). (B) Arrows show tumor cells which were positively stained with S100 (S100, 400×).

An otherwise healthy 2 years old male patient was consulted from pediatric emergency department with acute onset peripheral facial nerve palsy. According to the history achieved from his parents, he had no otalgia or otorrhea complaint. In the morning after he woke up, his parents realized that he could not close his left eye during crying. On his physical examination otoscopic findings revealed narrowing of the external ear canal from the posterior part by a mass originating from the mastoid bone. Tympanic membrane was hardly seen and a bluish color mass was present on the middle ear. He had left House–Brackmann grade IV peripheral facial nerve paralysis. Cranial tomography revealed a mass comprising left mastoid air cells, extending to the middle ear (Fig. 1). Magnetic resonance Imaging of the temporal bone demonstrated a solid, densely contrast enhancing hypointense mass which obliterates the mastoid air cells and extends into posterior part of inner ear without intracranial involvement (Fig. 2). Mastoidectomy and biopsy from the mass was performed under general anesthesia. Pathological investigation showed infiltration with tumoral cells having oval orthochromic nucleus. Immunohistochemical staining demonstrated positive staining with CD1a and S100 antigens (Fig. 3). The diagnosis was well consistent with LCH. After Pediatric Hematology and Oncology consultation, systemic evaluation was performed with abdominopelvic, neck, thoracic tomography and whole body bone scintigraphy. There were metastatic foci in mandibula and calvarium including parietal bone and vertex in bone scintigraphy. Seven cycles of chemotherapy regimen including Vinblastin (Vinko® 1 mg/mL. Koçak Farma drug-İstanbul-Turkey) and oral prednisolone (Deltacortil® 5 mg. Pfizer drug-İstanbul-Turkey) treatment were administered. In the control imaging studies there was half shrinkage of the primary tumor and there was decrement in radioactive Tc-99m intensity in mandibular and calvarium metastases. The patient's left facial paralysis was completely recovered after treatment. The patient is still followed up with Pediatric Hematology and Oncology department.

## Discussion

Langerhans cell histiocytosis is characterized by pathognomonic LC proliferation which are histiocytes normally found in dermis. Etiology of LCH is unknown, variable hypotheses such as neoplastic, immunologic or inflammatory processes are investigated but still there is no proof explaining the exact pathogenesis. LCH is not familial or hereditary, there is no race predominance. The disease is mostly seen in flat bones of children such as ribs, pelvis, scapula and skull. Temporal bone involvement is rare.^4^

When temporal bone involvement presents in LCH, a great suspicion is needed to correctly diagnose this rare disease, because sign and symptoms are non-specific and they mimic more common diseases of the ear such as suppurative otitis media, chronic otitis media, aural polyps, simple otitis externa and acute mastoiditis. Common presenting symptoms are postauricular swelling, chronic otorrhea, otalgia, hearing loss and bleeding. Involvement of the inner ear and sensorineural hearing loss are less common presenting symptoms due to compact nature of petrous apex and otic capsule.^4^

Differential diagnosis of pediatric peripheral Facial Nerve Paralysis (FNP) is broad including Bell's palsy, infections like acute otitis media, trauma, congenital anomalies and rarely malignancies. Most of the cases are acquired rather than congenital and number of cases is much less (5–21/100,000) when compared to adult patients. Tumors account for 2–12% of all pediatric facial nerve paralysis cases. The most common types are leukemias, teratomas, soft tissue sarcomas and neuroblastoma.^5^ Although LCH involves temporal bone in 19–25% of cases, with bilateral involvement in 1/3 of these ones,^1^ facial nerve paralysis is a very rare finding. In the series of Nicollas et al.^6^ with 42 patients, Surico et al.^2^ with 34 patients, McCaffrey et al.^7^ with 22 patients, Fernández Latorre et al.^8^ with 14 cases, Abdel-Aziz et al.^4^ with 12 patients, and Saliba et al.^3^ with 10 patients, none of the patients had facial nerve paralysis as initial symptom or in the course of the disease. In the most broad serie, almost 50 years ago, Tos^9^ presented only 2.8% of facial paralysis in 500 patients of LCH patients. In 1993 Goldsmith et al.^10^ presented a case of petrous apex LCH with FNP. The lower rate of facial nerve paralysis than expected for LCH cases with temporal involvement may be due to the tumor's having a lower affinity to the neural structures. Facial palsy can arise from compromised blood supply due to destruction of the osseous canal rather than direct invasion.^9^

For the diagnosis of LCH, pathological investigation of the biopsy specimen is mandatory. For biopsy achievement in cases of temporal bone involvement, a mastoidectomy is mostly required like our case.^3^ In the pathological investigation LCs have abundant vacuolated cytoplasm with vesicular oval intended nuclei. Light microscopy is insufficient for definitive diagnosis. Electron microscopy shows immature LCs with characteristic pentalaminar organelles called as Birbeck granules. But since electron microscopy is not cost effective, histopathological diagnosis is mostly achieved by immunohistochemical staining. Tumor cells are positively stained with surface antigen S-100 and leukocyte adhesion molecules CD1 antigens like in the case of our patient.^3^

For treatment options of temporal bone LCH cases there is a great variability between authors. Surgery, radiation therapy, chemotherapy and combination of these treatment options exist. In the current treatment options most of the authors agree that surgery must be chosen for tumors which can be totally excised. Chemotherapy is helpful in multifocal disease and must be preferred when surgical total removal could not be achieved.^3^ Stereotactic radiosurgery and intralesional corticosteroid treatment options are other treatment alternatives that can be applied.^4^ For the chemotherapy protocol stage of the tumor is important.^9^ According to LCH study group, LCH I protocol consists of vinblastine + prednisone, is applied for cases of single system-multifocal, or multisystem disease. LCH II protocol adds etoposide to LCH I protocol for multisystem disease with risk of organ dysfunction. LCH III protocol adds methotrexate to two drug treatment for cases of multisystem disease and organ dysfunction, the LCH I is the most widely used protocol.^4^ There is no globally accepted treatment option for all temporal bone LCH cases due to scarcity of number of patients. Since our case had a single system-multifocal tumor with temporal, calvarial bones and mandibular involvement, we preferred chemotherapy including LCH I protocol for our treatment option. Surgery was not performed except for biopsy procedure, since the tumor was not amenable to total excision. In the follow-up period, facial paralysis totally recovered with LCH I protocol chemotherapy for our case.

The prognosis of LCH is dependent on the stage of disease and age of the patient. Age lower than 2 years, multisystem disease and organ dysfunction are bad prognostic factors for LCH cases.^3^

## Conclusion

As a conclusion, for children with acute onset peripheral facial paralysis, langerhans cell histiocytosis must also be thought in differential diagnosis. Treatment plan must be made after imaging studies for determining widespread of the disease with a multidisciplinary approach including Otorhinolaryngologist, Pediatric Oncologist and Radiologist.

## Conflicts of interest

The authors declare no conflicts of interest.
